# Anyone Can Get Old—All You Have to Do Is Live Long Enough: Understanding Mortality and Life Expectancy in European Hedgehogs (*Erinaceus europaeus*)

**DOI:** 10.3390/ani13040626

**Published:** 2023-02-10

**Authors:** Sophie Lund Rasmussen, Thomas B. Berg, Helle Jakobe Martens, Owen R. Jones

**Affiliations:** 1 Wildlife Conservation Research Unit, Department of Biology, University of Oxford, The Recanati-Kaplan Centre, Tubney House, Abingdon Road, Tubney, Abingdon OX13 5QL, UK; 2 Department of Chemistry and Bioscience, Aalborg University, Fredrik Bajers Vej 7H, DK-9220 Aalborg, Denmark; 3 Naturama, 30 Dronningemaen, DK-5700 Svendborg, Denmark; 4 Department of Biology, University of Southern Denmark, 55 Campusvej, DK-5230 Odense M, Denmark; 5 Department of Geosciences and Natural Resource Management, Section for Forest, Nature and Biomass, Copenhagen University, 23 Rolighedsvej, DK-1958 Frederiksberg C, Denmark; 6 Interdisciplinary Centre on Population Dynamics (CPop), University of Southern Denmark, 55 Campusvej, DK-5230 Odense M, Denmark

**Keywords:** sex-biased longevity, age structure, wildlife conservation, age, matrix models, life tables, sex-biased mortality, European hedgehogs, periosteal growth lines, urban and rural

## Abstract

**Simple Summary:**

As populations of European hedgehogs are declining, it is vital that we monitor and understand the population dynamics of this species to optimise conservation initiatives to protect the hedgehogs in the wild. We determined the age of 388 dead European hedgehogs, collected by volunteers from all over Denmark, by counting periosteal growth lines, a method similar to counting year rings in trees. The overall mean age was 1.8 years (1.6 years for females and 2.1 years for males), and the range was between 0 and 16 years. We found the oldest scientifically confirmed hedgehogs in Europe among our samples (11, 13, and 16 years), with previous research recording a maximum age of 9 years. We constructed life tables showing life expectancies at 2.1 years for females and 2.6 years for males. We found that male hedgehogs were more likely to have died in traffic than females and that traffic-related deaths peaked in July for both sexes. For non-traffic deaths, most males died in July, and most females died in September. Most of the road-killed individuals in the study died in rural habitats. The degree of inbreeding did not influence longevity. These new insights may be used to improve future conservation strategies protecting the European hedgehog.

**Abstract:**

The European hedgehog is in decline, triggering a need to monitor population dynamics to optimise conservation initiatives directed at this species. By counting periosteal growth lines, we determined the age of 388 dead European hedgehogs collected through citizen science in Denmark. The overall mean age was 1.8 years (1.6 years for females and 2.1 years for males), ranging between 0 and 16 years. We constructed life tables showing life expectancies at 2.1 years for females and 2.6 years for males. We discovered that male hedgehogs were more likely to have died in traffic than females, but traffic-related deaths peaked in July for both sexes. A sex difference was detected for non-traffic deaths, as most males died in July, and most females died in September. We created empirical survivorship curves and hazard curves showing that the risk of death for male hedgehogs remains approximately constant with age. In contrast, the risk of death for females increases with age. Most of the collected road-killed individuals died in rural habitats. The degree of inbreeding did not influence longevity. These new insights are important for preparing conservation strategies for the European hedgehog.

## 1. Introduction

The life history strategy of a species—the age- or stage-specific patterns of events in a life cycle—is shaped by evolution to maximise fitness [1]. Variation across species and environments and the trade-off between reproduction and survival have led to a striking diversity in life history strategy (and its component traits) across the tree of life. For example, short-lived species such as small rodents tend to be precocious and prodigious breeders, while large-bodied mammals such as elephants and whales grow and reproduce slowly [2]. Similar patterns are also apparent within species: variation in the risk of predation, which alters the survival–reproduction trade-off, may result in shifts towards a “fast” strategy with a shorter generation time, higher reproduction, and shorter life span [3]. Understanding the life history strategy of a species is beneficial for developing robust species-specific conservation practices.

Population models are important tools in species conservation that are particularly relevant when a species appears to be in decline, as in the example of the European hedgehog (*Erinaceus europaeus*). Ecological population modelling can be used to quantify the relative importance of different parts of the life cycle for population growth to predict changes in parameters such as population size and age distribution and understand the impact of exogenous drivers [4]. Population modelling approaches have been widely applied in tackling the conservation of countless species, such as the Pacific fisher (*Pekania pennanti*) [5] and California spotted owl (*Strix occidentalis occidentalis*) [6], leading to increased political priority and conservation efforts [7,8]. These modelling efforts require parameter estimates of key factors such as survival probability and reproductive output at different life cycle stages or ages. For the European hedgehog, a species of growing conservation concern, there is still considerable uncertainty about these key traits. This lack of knowledge has so far hampered the construction of robust population models to explore the dynamics and conservation status of this species.

The European hedgehog is a widely distributed species that can survive across diverse habitat types in rural as well as urban landscapes [9,10]. However, recent research on national and local scales has documented a decline or indicated concerns for a decline in their populations in several western European countries [11,12,13,14,15,16,17,18,19,20,21]. The suspected reasons for the decline include the following: habitat loss; habitat fragmentation; inbreeding; intensified agricultural practices; road traffic accidents; a reduction in suitable nest sites in residential gardens, as well as biodiversity, and hence food items; accidents caused by garden tools, netting, and other anthropogenic sources in residential gardens; molluscicide and rodenticide poisoning; and, in some areas, badger predation [12,22,23,24,25,26,27,28,29,30,31,32,33,34,35].

Hedgehogs hibernate to conserve energy during colder periods during which food availability is low [36]. In Denmark, they usually hibernate from late September (male adults), late October (female adults), or mid-November (juveniles) to around mid-April or mid-May [33,37,38]. However, juveniles may extend their activity period until mid-December if the weather conditions are mild, leaving food items such as slugs, snails, and insects available [33]. The period of winter inactivity is reduced for European hedgehogs residing in milder climates, such as Southern Europe and New Zealand. In New Zealand, hedgehogs may even stay active throughout the year [36]. Hibernating hedgehogs experience periodic arousals every 7–11 days on average, and during these periodic arousals they may remain active for a few days, leaving the nest to forage, or simply change nests [10]. Up to eight nest changes during a Danish winter have been recorded [33].

During hibernation, when the calcium metabolism in the hedgehog is modified, bone growth is reduced markedly or even stopped. This causes densification of the bone resulting in periosteal growth lines, or lines of arrested growth (LAGs), which are formed in the periosteum of the bones. The periosteal growth lines appear to be caused by the arrest of cartilage growth, leading to the infusion of the cartilage plate with apatite (calcium phosphate) [39]. In general, growth lines are developed in vertebrates as the metabolism and growth are inhibited by seasonal cycles in the environment [40]. They are comparable to tree annual growth rings and become visible in stained sections of bones such as the mandible, the lower jaw [41,42]. This phenomenon allows researchers to count the number of hibernations a hedgehog has survived. However, bone growth or bone deposition in the periosteum may also become reduced during periods of stress, potentially forming accessory lines in the bone, which may complicate the interpretation of growth lines for age determination [41]. Previous studies on growth lines in mammals, or LAGs, from known-age individuals indicate that the number of LAGs in bones positively correlates with age [43,44,45], though the evidence base is larger for herpetofauna [46].

Data on age-specific survival and reproduction aid the parameterisation of matrix models providing insight into the population dynamics of hedgehogs. For hedgehogs, one approach to accommodate the demand for such age-related data is to conduct age determination studies on deceased individuals. Previous research into the age structure of European hedgehogs is based on four different methods: capture–mark–recapture, counting of periosteal growth lines in the mandible of the hedgehog, measuring the extent of tooth wear (tooth abrasions), and estimating the number of growth lines in the teeth, typically in the molar cement of the M^1^ [41,42,47,48,49,50,51,52,53,54,55,56,57,58,59,60,61,62,63,64]. As an alternative method for determining the life expectancy of hedgehogs, Parkes [65] used a formula by Petrides [66] to calculate an average life expectancy of 1.97 years based on a sample of 144 individuals divided into adults and juveniles (N = 73). Morris [67] also developed a method for age determination in hedgehogs by using X-rays to measure epiphyseal fusion in the forefoot, as the presence of epiphyseal cartilage in the metacarpal bones is a juvenile characteristic. This method classifies juvenile hedgehogs into four age categories and distinguishes juvenile hedgehogs from adults. Table 1 provides an overview of the data from the present and previous age determination studies on hedgehogs [36,41,42,47,48,49,50,51,52,53,54,55,56,58,59,60,61,62,63,64,65,68].

Our aim with this study was to add to this information by collecting and analysing a substantial dataset, including data on inbreeding, which have hitherto not been included in previous age structure studies on European hedgehogs, providing the opportunity to study a combination of longevity, inbreeding, and cause and timing of death in the Danish hedgehog population. We obtained our data from a large sample of deceased Danish hedgehogs collected via a citizen science study. Specifically, we investigated the ages of deceased individuals collected from the Danish hedgehog population by counting periosteal growth lines in transverse sections of the mandibles and creating life tables based on these data. We also explored the timing and cause of death in our sample to understand whether sex, season, or habitat type (urban/rural) influenced the cause and frequency of death. Finally, we aspired to understand whether the degree of inbreeding influenced longevity in European hedgehogs. The research was performed to achieve a deeper understanding of the population structure and specific factors influencing hedgehog ecology and to provide data that improve the conservation initiatives directed at this declining species.

## 2. Materials and Methods

We obtained samples of dead hedgehogs from the Danish public as part of the citizen science project “The Danish Hedgehog Project”, which aimed to use dead hedgehogs to assess the general health of the Danish hedgehog population. The public outreach was primarily based on >200 features in the national and local media from April to December 2016. With approximately 400 volunteers from throughout Denmark collecting 697 dead hedgehogs, during May to December 2016, there was an excellent geographical representation of the entire country’s population. The hedgehogs were primarily road kills, but the sample set also included individuals dying in care and individuals dying from other causes in the wild.

For each specimen, we collected geolocation (latitude and longitude), sex, cause of death, and date of death. We also took tissue samples (skin, fat, and muscle) for genetic analysis to quantify inbreeding. The individuals were in different stages of preservation, and many of the road-killed individuals were not intact. However, heads, front legs, and any organs present were extracted for future research. Out of the 697 dead individuals, 388 were adequately intact to have mandibles of a sufficient quality to use for age-at-death determination.

To determine the age-at-death of the hedgehogs, we counted periosteal growth lines in transverse sections of the mandibles, with each growth line indicating one hibernation season, as described by Morris [41]. To do this, we first macerated the bones to obtain clean skeletal remains. This was achieved by placing the hedgehog heads and legs in gauze with knots between the body parts and on the ends and placing the gauze packs in a bucket of water with a constant temperature of 40 °C for 10 days in a heating cabinet. After 10 days, the water was changed three times at 2–3 h intervals. Then the gauze packs were boiled for 10 min at 90–95 °C in clean water, followed by a drying period of 5 days. After drying, the remaining fat on the jawbones was removed with a cotton bud. Some samples were placed in clean water supplemented with 1:100 H_2_O_2_ for 30 min after boiling, after which the water was changed again. After 24 h in the water, the samples were then removed and left to dry before the bones were ready for decalcification.

To decalcify the mandibles, the samples were first placed in 2 cl glasses and covered in 10% formalin for 24 h. Afterward, the jaws were rinsed in running water and put in 2 cl glasses with water for 24 h. Finally, the mandibles were rinsed in running water and stored in 2 cl glasses with Rapid Decalcifier (diluted 2:1, with two parts distilled water and one part decalcifier) added. The mandibles were removed from the decalcifier whenever they were bendable and cuttable with scalpel knives. The jawbones were checked regularly with 30 min intervals. Smaller mandibles from juveniles were only left in the decalcifier for 2 h; the larger ones were left for up to 9 h. On average, 4–5 h was sufficient, with a room temperature of 15 °C, even though previous studies describe a mean time of 6 h using the same product [50]. When the bones were sufficiently decalcified, they were rinsed in water, stored in plastic cups with screw lids, and covered in formalin ready for slide preparation.

We prepared the slides by taking an approximately 10 mm transverse section of the jawbone near the last molar with a scalpel knife and mounting it on a cutting plate with a drop of Optimal Cutting Temperature (O.C.T.) Compound (polyvinyl alcohol <11%, Carbovax <5%, nonreactive agents >85%), with the weakest part of the bone pointing upwards. The section was placed in the cryostat microtome (Leica CM3050 S) to be sufficiently frozen before cutting, making it as hard as possible, lasting approximately 3–3.5 min. The jawbone sections were then sectioned in the cryostat microtome at a temperature of −20 °C with a thickness of 50 μm.

The 50 μm jawbone sections were placed on SuperFrost Plus slides, and distilled water (Milli-Q) drops were added on top of the sections to keep them from drying out.

The water was then removed with an automatic pipette, and the sections were stained with crystal violet (0.005%) for 5 min, after which excess dye was removed with an automatic pipette. Distilled water (Milli-Q) drops were then added to rinse away the dye; afterward, the water was removed with an automatic pipette. Subsequently, the slide was coated with the mounting agent Aquatex to preserve the sample, and a cover slip was added on top of the slide. Compared to previous studies using the mounting agent Euparal, our samples did not require suction, but could be placed under the microscope immediately, and were completely dry and ready for age determination within 24 h.

To estimate the age-at-death, we analysed the prepared and mounted sections under a microscope at 20× objective giving a 200 times enlargement (Leica DM 5000B Fluorescence Microscope). A Canon EOS 1200 D Camera was connected to the microscope and was used for image recordings. Scientific Focus Acquisition Software 1 was used to view and edit the pictures. Contrast adjustments were carried out to improve the clarity of images. Final image processing and cropping and mounting of the images were performed with Adobe Photoshop CS5 and Illustrator CS5. We were then able to estimate age by counting the periosteal growth lines in the prepared transverse section of the lower jaw. At least two biologists evaluated each sample, counting the periosteal growth lines, and the results were compared. In the few cases of disagreement, the slides were reviewed and discussed, and a third observer was involved when needed. Discontinued and imperceptible lines were not included in the counts. After applying these methods, we had a full representation of the age structure in our sample of the Danish hedgehog population.

We analysed the age-at-death records, alongside covariates of habitat type, timing and cause of death, and sex. Our analyses of the timing of death and cause of death were restricted to individuals of known sex. We divided cause of death into two categories: traffic- and non-traffic-related deaths. Our analyses and figure preparations were performed in R [69].

To allow us to explore the influence of habitat on mortality, we classified each hedgehog sample into “urban” or “rural” habitat types. We did this for each geolocated sample based on land use types within a 500 m radius around the spot where the hedgehog was found. This area is roughly equivalent to a large hedgehog home range [10]. We obtained the land use data from the EU CORINE land cover dataset, which has a 100 × 100 m resolution (CLC 2012, Version 18.5.1), which resulted in 81 squares for each hedgehog. The CORINE data use satellite imagery and post-processing to assign habitat types as artificial surfaces, industry, agricultural areas, forest and semi-natural areas, wetlands, and water bodies. We then analysed the data using the raster package [70] in R [69] and reclassified the habitat types as “urban”, “rural”, or “other”, following the method of Rasmussen, et al. [71] (S1). We categorised individual hedgehogs as “urban” or “rural” based on the percentage representation of the two categories among 81 squares per individual. Combined with the information on the sex of the individuals killed in traffic, we investigated whether habitat type (urban or rural) or sex influenced the likelihood of being killed in traffic.

In a previous publication, we estimated inbreeding as the degree of individual heterozygosity (iH_O_) for a subsample of 151 aged individuals (78 males, 50 females, 23 of unknown sex) [31]. We tested whether inbreeding was associated with age-at-death using GLMs with quasi-Poisson error structure. We included the integer estimated age-at-death as the response variable and the inbreeding coefficient as the explanatory variable. We did this for both sexes combined, and for each sex independently.

We used quasi-Poisson GLMs to explore the association between age-at-death and inbreeding (degree of individual heterozygosity (iH_O_)), cause of death (traffic/non-traffic), and the interaction between them.

Lastly, to assess how the mortality risk (probability of death) changed with age, we constructed empirical actuarial life tables using the age-at-death information obtained from the dental analysis described above. We did this for males and females separately and excluded data from individuals of unknown sex. We used standard life table approaches and nomenclature [72,73]. Using these methods, we calculated empirical survivorship and hazard trajectories for both sexes.

## 3. Results

We determined the age-at-death of 388 hedgehogs by counting the periosteal growth lines in prepared transverse sections of the lower jaws (Table 2). The sample set consisted of 109 females, 177 males, and 102 of unknown sex. The mean age-at-death was 1.8 years (22 months) ± 95% CI [1.62, 2.04], distributed between the ages of 0 and 16 years (Table 2). The mean age-at-death was 1.6 years ± 95% CI [1.32, 1.93] for females and 2.1 years ± 95% CI [1.73, 2.47] for males. Dividing the individuals into categories based on the cause of death (traffic, dying from other causes in the wild, in-care), the mean age-at-death was 2.1 years ± 95% CI [1.80, 2.32], 2.0 years ± 95% CI [1.51, 2.54], and 1.3 years ± 95% CI [0.75, 1.83], respectively.

**Figure 1:**
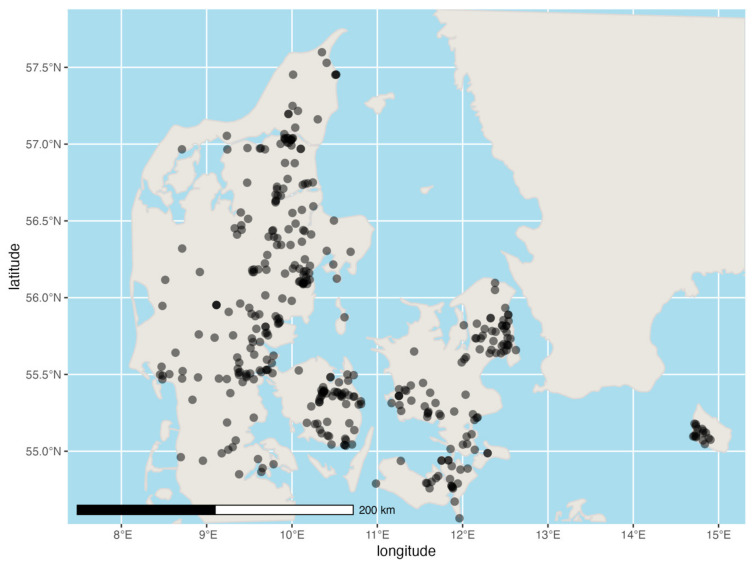
The distribution of the 369 geolocated samples collected during the study. Each point represents a single sample. Points are opaque and thus multiple samples from the same or nearby locations result in darker points.

Although the samples collected had a wide geographic coverage across Denmark, they were not evenly distributed (Figure 1).

**Figure 2:**
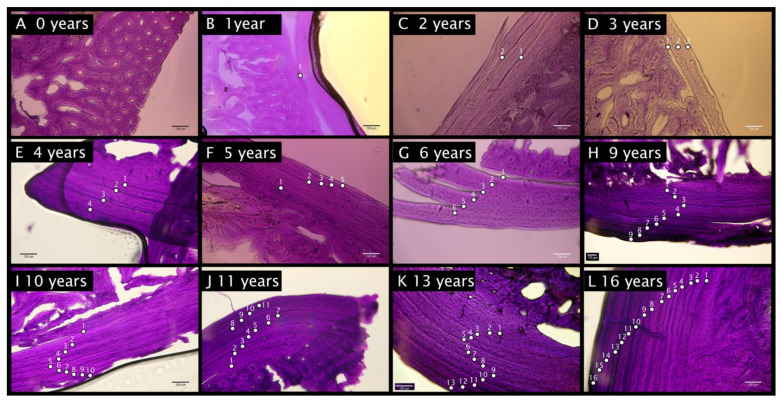
Illustrated examples of the 12 age categories observed in prepared transverse sections of the lower jaws of European hedgehogs from Denmark. Each white dot shows a growth line, and the small numbers next to each point indicate the numbers of growth lines, and their order, counted in each section. The age categories ranged from 0 years (dying before first hibernation), labeled A, to 16 years, labeled L.

Figure 2 presents illustrated examples of the twelve age classes determined in the present study.

### 3.1. Timing and Cause of Death

**Figure 3:**
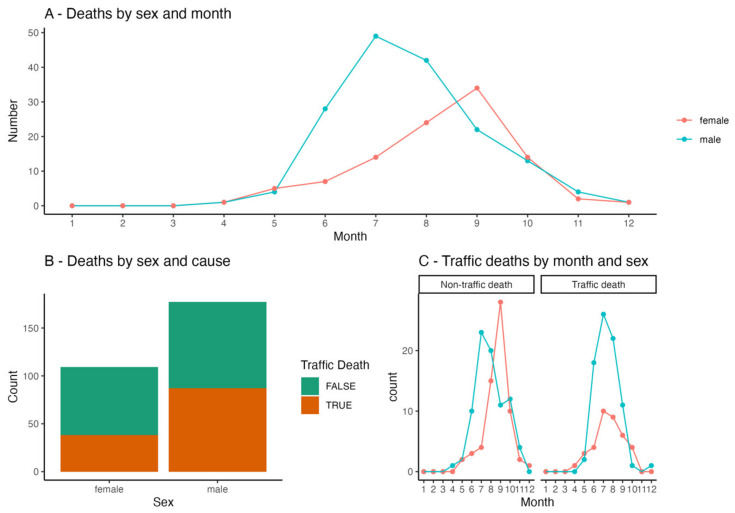
Proportion of deaths across the months of the year, disaggregated by sex (**A**): red colour represents females and blue colour represents males. Cause of death disaggregated by sex (**B**): orange colour is for traffic deaths and green colour is for non-traffic deaths (in-care, dying of other causes in the wild). Deaths disaggregated by both month and sex (**C**): red colour represents females and blue colour represents males.

Males accounted for 45.6% (177) of the samples, and females accounted for 28.1% (109). The remaining 26.3% (102) of the samples could not be sexed. Out of the 388 individuals, 55.7% (216) were road kills, 22.2% (86) died in care, 21.6% (84) died of natural causes in the wild, and 0.5% (2) could not be categorised due to unknown causes of death. The time distribution of samples through the year showed a clear modal distribution. The timing of the peak in deaths in general varied between the sexes, with the deaths of males being most prevalent in July and the deaths of females being most prevalent in September (Figure 3A). We divided causes of death into two categories: road traffic death and non-traffic-related death. There was a clear sex difference in this cause of death, with male samples being more likely to have died in traffic than females (Figure 3B). A sex difference was detected in the timing of the “peak death” month for non-traffic-related deaths (Figure 3C). For non-traffic-related deaths, males peaked earlier (July) than females (September), but for traffic deaths, the peaks were synchronous (July).

### 3.2. The Influence of Habitat Type (Urban/Rural) and Sex on the Amount of Road-Killed Individuals

**Figure 4:**
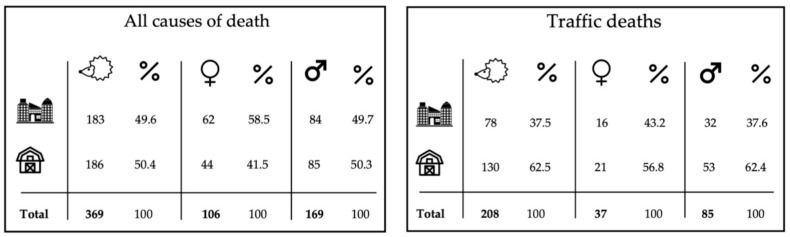
An overview of the dataset showing whether the 369 hedgehogs with geolocations died in rural (barn symbol) or urban (high-rise buildings symbol) habitats, divided into categories of all sexes, including the individuals of unknown sexes (hedgehog symbol), and each separate sex, respectively, including percentage representation of the 208 geolocated traffic-related deaths for each sex and habitat type (urban/rural).

Of the 369 dead individuals with precise geolocation information, 49.6% (183) were found in urban habitats and 50.4% (186) were found in in rural habitats, with a further relatively even distribution of individuals dying in urban or rural habitats when categorised into males and females (Figure 4). Of the 206 road-killed individuals with known geolocations, 37.5% (78) died in urban habitats and 62.5% (130) died in rural habitats, with approximately the same pattern showing when looking at the sexes separately.

### 3.3. The Effects of Genetic Heterozygosity on Age-at-Death

**Figure 5:**
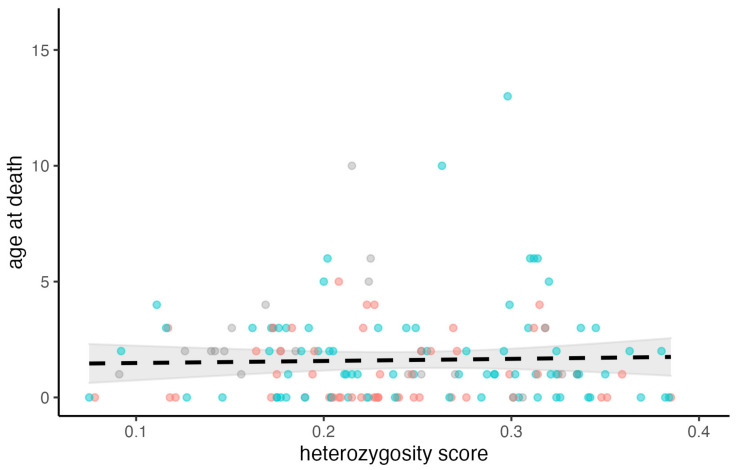
The relationship between individual heterozygosity (iH_O_) and age-at-death of the hedgehogs studied (N = 151) is not statistically significant. The red points represent females, the blue points males, and the grey points unknown sex. The line and ribbon represent the fitted model without sex included (sex does not influence the fit).

The mean inbreeding score, observed individual heterozygosity (iH_O_), was 0.240 (SD = 0.074), which indicates a moderate degree of inbreeding. There was no significant association between inbreeding and age-at-death (all animals: quasi-Poisson GLM, t = −0.344, residual d.f. = 149, *p* = 0.798; males only: quasi-Poisson GLM, t = 0.272, residual d.f. = 76, *p* = 0.785; females only: quasi-Poisson GLM, t = −0.271, residual d.f. = 48, *p* = 0.786) (Figure 5).

### 3.4. Exploring the Association between Age-at-Death and Degree of Inbreeding, Cause of Death (Traffic/Non-Traffic), and the Interaction between Them

The results showed no significant interaction between the cause of death and the degree of inbreeding (quasi-Poisson GLM: t = −0.321, d.f. = 147, *p* = 0.749), indicating that the effect of inbreeding was independent of the cause of death. Although the age-at-death tends to be a little higher in hedgehogs killed in traffic (1.972 years; 95% CI [1.667–2.277]) compared to those dying by other causes (1.645 years; 95% CI [1.333–1.957]), it is not significantly so (quasi-Poisson GLM: t = 1.481, d.f. = 386, *p* = 0.139).

### 3.5. Life Table Analyses

The life table calculations for males (Table 3) and females (Table 4) showed that life expectancy at age 0 (*e_x_*) was 2.6 years for males and 2.1 years for females, indicating that newborn male hedgehogs can expect a 24% longer life than females.

**Figure 6:**
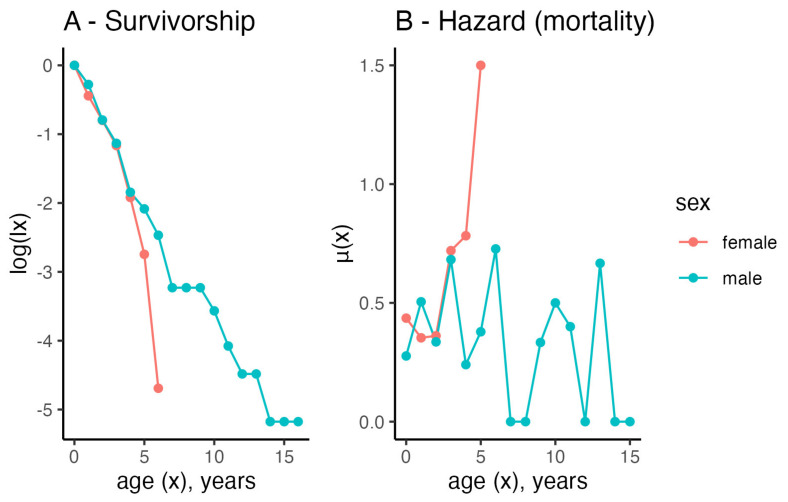
Empirical survivorship curves (**A**) and mortality rates (**B**) for male and female hedgehogs, calculated using standard life table methods.

The empirical survivorship and hazard trajectories (also known as mortality rate or death rate curve) showed a classic Type II survivorship for males and a Type I survivorship curve for females (Figure 6A). Type II survivorship is indicative of a constant risk of death with age while Type I is indicative of an increasing risk of death with age (Figure 6B), indicating that the risk of death in males is approximately constant despite increasing age and the risk of death in females increases with age.

## 4. Discussion

This research elucidated several interesting results describing the population dynamics of European hedgehogs, which will be applicable for the preparation of future conservation strategies for the declining population of this species. By counting the periosteal growth lines and creating life tables, we found a mean age for all individuals of 1.8 years, 1.6 years for females and 2.1 years for males, as well as 2.1 years for females and 2.6 years for males, respectively, the latter measured as the life expectancy at age 0, indicating that male hedgehogs live longer than females. We found that male hedgehogs were more likely to have died in traffic than females but that traffic-related deaths peaked in July for both sexes. A sex difference was detected in the timing of the “peak death” month for non-traffic deaths, as males peaked earlier (July) than females (September). Based on the life tables, we created empirical survivorship curves and hazard curves indicating that the risk of death in males is more or less constant despite increasing age and the risk of death in females increases with increasing age. We found a 50/50 distribution of hedgehogs dying in urban and rural habitats, with a further relatively even distribution of individuals dying in urban or rural habitats when categorised into males and females. Focusing on road-killed individuals, we found that approximately one-third of the individuals (37.5%; 78) died in urban habitats and two-thirds (62.5%; 130) died in rural habitats, with roughly the same pattern showing when looking at the sexes separately. Finally, we detected no significant association between the degree of inbreeding and age-at-death or between the degree of inbreeding and cause of death.

### 4.1. Periosteal Growth Lines

Periosteal growth lines are excellent instruments for age determination, but the method comes with certain caveats which should be considered when interpreting results [45]. Firstly, it is important to be aware that some growth lines may not represent hibernation but perhaps a period of stress for the animal, where the bone growth may be reduced or stopped altogether [74]. These so-called accessory lines tend to be less visible than hibernation lines after the staining of the jawbone sections. Secondly, the growth lines may be absent in cases where the individuals do not hibernate. This statement is supported by Morris [41], who noted that a young hedgehog kept active and well-fed throughout winter, and which did not hibernate, failed to form any growth line in the bone. This means that the assessment of periosteal growth lines is not appropriate for age determination in hedgehogs that do not hibernate, such as many individuals in New Zealand [75]. This problem may be increasingly relevant in other populations too. For example, in recent years during exceptionally mild winters, hedgehog rehabilitators in the UK and Italy have recorded much more activity among hedgehogs during periods where they were supposed to be hibernating.

It is currently unknown how much activity it takes to prevent the growth lines from forming and whether additional growth lines could form when individuals experience interrupted hibernation (e.g., if an individual’s winter hibernation is split into two hibernation periods by an active period caused by disturbance). Because these factors may compromise the validity of the age determination method, it is important to keep them in mind when interpreting the results. In the current Danish climate, it would be highly unlikely that hedgehogs would remain active throughout winter, and to our knowledge non-hibernation in these animals has never been recorded in Denmark, despite ad libitum winter food availability at local feeding stations in residential gardens. Studies of hibernation in Danish hedgehogs have failed to provide evidence of extended periods of activity between multiple distinct periods of dormancy [33,38,76,77]. Nevertheless, it is possible that during recent years with the extremely mild winter conditions, and therefore periods of potential food availability, the hedgehogs may have become active for longer periods in Denmark as well. The current study benefitted from having a large sample size consisting mainly of individuals aged 0 and 1 years (n = 109 and n = 115, respectively) with known dates of death. In addition to the jaw bones being markedly smaller than those of adult hedgehogs, we could simply cross-check the growth line count with the age-at-death determined by other means. For example, we know that juveniles are almost all born in late July or during August in Denmark and that females may produce second litters which are born from September to late October [33]. This clearly indicated that small individuals with no visible growth lines, killed in the autumn of 2016, were certainly less than one year old. In the cases where a single growth line was detected, we studied the death date of the individual to determine whether it had reached the age of one year (death before or after June 2016). In none of the cases where we counted growth lines in small jaw bones, which were easily recognisable as being from juveniles or subadults, did we detect more than one clear growth line. Therefore, based on this knowledge, we have concluded that hedgehogs in our study were unlikely to have formed accessory growth lines during the hibernation period. However, what we cannot conclude is whether the reason was a lack of activity during winter or that they simply did not form extra growth lines in cases where the hibernation had been interrupted for a longer period of time. Further research of the dormancy period required for bone deposition to become slow enough to allow the formation of growth lines is required.

Our estimate of a mean age-at-death of 1.8 years is roughly equivalent to the findings of previous studies (presented in Table 1), ranging from 0.4 years to 3.3 years with a median of 2. However, several of these studies excluded individuals aged <1 year. If the individuals <1 year (109) were excluded from the present dataset, the mean age-at-death would be 2.5 years (279). Additionally, it should be noted that the previous studies used four different age determination methods, which could lead to a variation among results. Furthermore, the method of age determination would suggest that we have recorded the oldest confirmed European hedgehogs among our samples (11, 13, and 16 years), with previous research discovering a maximum age of 9 years [50]. This could be explained by our larger sample size (388) compared to the range of 62–244 individuals in previous studies (see Table 1), with a median of 97 hedgehogs and a mean of 112 hedgehogs ± 95% CI (87,136)

### 4.2. Timing and Cause of Death

Although our study used a large sample size of dead hedgehogs collected by volunteers throughout the whole of Denmark from May to December 2016, we must be aware of the potential bias in the distribution of roadkill per calendar month, because the public awareness of the project gradually built up as more news features were launched during the year. There is also potential bias in the cause of death among the hedgehogs because the road-killed individuals are easier to find than those with natural death and did represent a majority of the sample size.

Our results indicate that male hedgehogs are more prone to death in traffic than females, especially during the mating season, which is usually in June and July, but sometimes extends into August, in Denmark [33]. This is explained by the fact that males tend to have larger home ranges than females [10,33,36] and likely move over larger areas bringing them into contact more frequently with roads and road traffic. Previous research has also demonstrated that male hedgehogs tend to cross roads more often than females [29]. It is notable that the home ranges of males, but not females, increase during the mating season [33], which would exacerbate the male–female difference. However, traffic deaths were also higher for females during July and August, which includes the mating season, probably because they also tend to move over relatively larger areas in the search for mates, although not to the same degree as males [10,33,36]. Females may increase movements, and thus road-crossing behavior, during these periods to either search for mates or forage more widely during lactation. Previous research on traffic deaths of Danish wildlife in 1957–1958 and 1964–1965 [78], showed similar patterns: of 178 road-killed hedgehogs, most deaths occurred in October (66, i.e., 37%), and counts from July to September showed approximately 30 individuals per month (17% per month). This means that collectively July–October was the most dangerous time of the year for hedgehogs. These earlier results contrast with the present study where we found that road-kill deaths were concentrated in June–September, with a clear peak in July. A possible explanation for this difference between the studies could be that only a few road-killed hedgehogs were counted in May during the years of 1957–1958 and 1964–1965 (3), indicating that the hedgehogs may have become active after hibernation considerably later during those years compared to 2016, when the samples from the present study were collected, and that this delay could have influenced the results. Furthermore, Hansen [78] was limited to a local scale compared to the present study which represented road kills from all over Denmark. In comparison, Holsbeek, et al. [79] also found a peak in the number of road-killed hedgehogs during July, in Belgium, with a large sample size of 1281 road-killed hedgehogs in 1995–1996. Another possible influence potentially adding to the peak in road-killed individuals collected during the summer could be the longer daytime hours, making the hedgehog cadavers more visible to the volunteers collecting them.

Of the non-traffic-related deaths, incidences peaked in July for males and August and September for females, which is likely explained by the physical exhaustion caused by the mating season leaving males more vulnerable to infections, starvation, and dehydration at this time. This is evidenced by the fact that male hedgehogs are commonly brought into care during this period. There was a clear increase in non-traffic-related deaths among females in August and September. This is likely associated with greatly elevated energetic costs during lactation and compounded by parental care responsibilities and, consequently, the associated reduced time allocated to foraging. In September, the higher death rates could also be explained by a reduced body, and general health, condition after raising the young.

### 4.3. Testing the Influence of Habitat Type (Urban/Rural) and Sex on the Number of Road-Killed Individuals and Using Data on Road-Killed Individuals for Population-Level Research

Monitoring patterns and trends in road-kill data can be useful for many ecological purposes including tracking population trends, mapping native and invasive species distributions, studying animal behaviour, and monitoring pollutants and wildlife disease [80]. In European hedgehogs, longitudinal studies have highlighted a drastic population decline in the UK [20,81]. Estimating and understanding the risk of death experienced by individual animals requires a carefully planned and well-controlled study. It is well known that road-kill data include potential sampling biases as simple counts of dead individuals do not provide valid information about the probability of hedgehogs being killed in traffic. Risk calculations require both the numerator (number killed) and denominator (number exposed to risk). Nevertheless, the spatial pattern of road-kill risk is certainly closely related to hedgehogs’ live distribution, indicated by significant live distribution–road-kill overlap in a UK study [82].

Another potential bias in our dataset is the relatively large proportion of males in our sample of road-killed individuals. Unfortunately, we are not aware of the spatial distribution of males versus females, so this pattern could be caused by there being more males in the sampled populations. Alternatively, it is possible that males are more prone to taking risks such as crossing roads [83]. Male hedgehogs have larger home ranges than females [84,85,86], and their home ranges appear to be larger in rural areas compared to urban [33], so by traveling further the males may be more exposed to being killed on roads, especially in landscapes that are heavily fragmented by roads. This line of thinking supports the results from previous studies on hedgehog road casualties, where a higher proportion of males were recorded [87,88,89], and is consistent with our own dataset.

The tendency in our dataset for rural areas to have a higher proportion of road-kill deaths is interesting. There are numerous potential explanations for this observation. It could for example be due to a sampling bias whereby rural cadavers may have remained intact on rural roads that have lighter traffic [90], leaving them easier to collect. Alternatively, our result could be explained by differences in road crossing behaviour between urban and rural areas. This idea is supported by previous studies, which have shown that hedgehogs tend to cross smaller and less busy roads more frequently, leading to a higher proportion of hedgehogs being killed in traffic on the smaller rural roads [29,88,89,91,92,93]. Kent, Schwartz and Perkins [82] also found that hedgehog road-kill risk was reduced at high urbanity levels. Other factors, such as speed of traffic, type and width of the road, and density of scavenger species that could move the carcass before collection, may all have influenced the results in terms of the distribution of road-killed individuals between urban and rural habitats. Slater [94] documented that simple counts of wildlife corpses found on roads are severe underestimates of the actual road casualty rate, with death rates up to 12–16 times that observed by simply counting corpses, due to the removal of carcasses by scavengers. The removal rates depend on different factors such as species of scavengers, road structure, road traffic, season, time of day, and weather conditions. In a study on the influence of scavengers on road-kill data, Ratton, et al. [95] found that 89% of carcasses were removed by scavengers in the first 24 h and 66% were removed within 12 h. This could potentially have caused a sampling bias in our dataset, as scavengers increasingly tend to inhabit urban areas [96]. Other factors which may have influenced the sampling of the present dataset through citizen science could be the number of people that would see the hedgehog carcass and the probability that they would collect the carcass, which is contingent upon the ease of collection related to road type, safety, and the state of the carcass after being run over. Lastly, several factors intrinsic to the hedgehog population could also have influenced the sampling, such as population density, population structure (age/sex), and behaviour including avoidance and acclimatisation to traffic. When considering all these different factors, it would be possible to invent many scenarios that would produce the observed results, even if there were no differences in individual risk between rural and urban settings.

It is a general challenge to properly interpret data from sample sets of wildlife casualties, as there will always be biases connected to the collection of samples, not only for road-kill data. Another example could be bycatch and stranding data of marine mammals, because one must ask whether the sample is representative of the wild population in general and whether there is a tendency for individuals of certain categories of, e.g., age, sex, or even degree of boldness to become stranded. Regardless of the limitations mentioned, the conclusion must be that it is still preferable to work with the available data compared to having no research at all, and to interpret these datasets with caution, being aware of and articulating the potential caveats when presenting the results.

### 4.4. The Effects of Genetic Heterozygosity on Longevity

From a conservation perspective, it was a positive discovery that the degree of inbreeding did not seem to influence the longevity of European hedgehogs, because previous research has found that Danish hedgehogs have low genetic heterozygosity [31,32], which is indicative of a high degree of inbreeding. A high degree of inbreeding could lead to inbreeding depression including hereditary, and potentially lethal, health conditions in the hedgehogs [97].

### 4.5. Exploring the Association between Age-at-Death and Degree of Inbreeding, Cause of Death (Traffic/Non-Traffic), and the Interaction between Them

We found a tendency for age-at-death to be a little higher in hedgehogs killed in traffic compared to hedgehogs dying from other causes (in-care, natural causes in the wild), but the results were not significant. This difference could be influenced by the large number of orphaned juvenile hedgehogs dying in care in the sample set, constituting 59% (51/86) of the individuals in the category “dying in care”. Furthermore, males in the study were more likely to have died in traffic and showed a higher age-at-death than females, which could also have influenced the results.

### 4.6. Life Table Analyses and the Life Expectancy of Females and Males

Our life table analyses showed that life expectancy at age 0 (*e_x_*) was 2.6 years for males and 2.1 years for females. During the manual calculation of periosteal growth lines, we also found that all individuals >6 years were males. In the age determination study, the mean age was 1.8 years, which is lower than the results of the life table analyses. This is likely caused by the inclusion of a large number of individuals of unknown sex in the age determination study (27/109 individuals aged 0 years and 40/115 individuals aged 1 year out of a total sample size of 388 individuals) compared to the life table analyses only including individuals of known sex.

Our study revealed that male hedgehogs are more prone to death in traffic than females, especially during the mating season, but they also appear to live longer than females. This is surprising given that road traffic is thought to be a major cause of hedgehog deaths [25,87,98]. The mean age-at-death of road-killed individuals was higher (2.1 years) than that for the categories of non-traffic deaths (2.0) and individuals dying in care (1.3). Therefore, it is not surprising that males (87, 69.6%), which were more likely to be road-killed than females (38, 30.4%), also had a higher age-at-death than females. A large proportion of the hedgehogs dying in care are orphaned hoglets, which likely explains the lower age-at-death for this category of individuals.

Previous research on the life span of European hedgehogs has had mixed findings, with some studies of relatively balanced, or equal, male–female ratios showing that females have a longer life span [50,51,52,54] and others concluding that males live longer [49,53,55,61]. One possible explanation for the difference in life expectancy between male and female hedgehogs in the present study could be the larger sample size of males compared to females, which may have introduced a bias by increasing the likelihood of longer-lived individuals in the larger sample of males.

However, with these potential biases set aside, the finding that males live longer than females is surprising, because females of other vertebrate species, including humans, tend to live longer than males [99,100]. Clutton-Brock and Isvaran [100] found that reduced longevity in adult males compared to females is common in polygynous vertebrate species due to an earlier onset and more rapid progression of senescence in males, with a possible explanation for this being the intense intra-sexual competition for breeding opportunities between males in polygynous species. This is supported by Trivers [101], suggesting that the higher mortality of males in polygynous mammals is driven by the larger potential reproductive benefits of winning competitive encounters for males than females, and that the behavioural traits which are thought to enhance competitive success usually trade off against survival. According to Clutton-Brock and Isvaran [100], the magnitude of sex differences in life expectancy is determined by the degree of sex differences in the duration of effective breeding (DEB), defined as the period over which individuals are likely to breed successfully, causing the sex differences in aging and adult longevity to be more pronounced in polygynous species. However, DEB does not seem to be lower in male European hedgehogs compared to females. Furthermore, European hedgehogs are polygynandrous, as both males and females mate with several partners [10,36], which means that the males are not territorial and have no harems to protect, decreasing the intra-sexual competition compared to polygynous species. The male hedgehogs do occasionally fight each other for the right to mate with a female, which can lead to injuries and other fitness costs. However, at the same time, the hedgehogs are solitary, and the males do not take part in the rearing of offspring [10,36], which could potentially give the males an important fitness advantage compared to the females. In badgers, *Meles meles*, another polygynandrous mammal species, which are intraguild predators of, and share habitats with, European hedgehogs [26], males show higher body mass senescence rates compared to females [102]. The authors found that this sex difference was largely caused by the negative fitness effects of the intensity of intra-sexual competition in males, especially that experienced during early adulthood. Male European badgers explore mating opportunities both within and among social groups, which leads to a high rate of extra-group paternity [103] with a higher incidence of bite-wounding and mortality among males, suggesting that the intra-sexual reproductive competition is more intense among males than females [104]. Macdonald, et al. [105] detected no significant sex-bias in badger mortality rate, and Sugianto, et al. [106] found that badgers showed similar senescence schedules (somatic and hormonal) between the sexes, in the same study population. This difference in longevity among sexes compared to hedgehogs could therefore be explained by the fact that male badgers experience higher levels of intra-sexual competition, often live in complex social groups, and appear to take part in the rearing of the young, with some female individuals even showing alloparental behaviour [107]. These contrasting factors compared to hedgehogs highlight our previously mentioned possible explanations for the male-biased difference in longevity in hedgehogs.

### 4.7. Life Expectancy, Risks, and the Effects on Breeding

Our counting of periosteal growth lines and the life table analyses showed slightly different results. Our analysis using periosteal growth lines showed a mean life expectancy of 1.8 years overall (including individuals of unknown sex), with 1.6 years for females and 2.1 years for males. Discovering that the hedgehogs, on average, reach an approximate age of two years in Denmark is positive in the sense that these individuals will have the opportunity to take part in two mating and breeding seasons during their life time, since most individuals will become sexually mature during their second summer (aged one year) [10,36]. This is an important insight into the population dynamics of hedgehogs, as this will in theory positively influence the population growth rate and hence the survival of the population.

We saw a high proportion of individuals dying at the age of one year (n=115/388, 30%). Fortunately, our data also showed that if the individuals survived this life stage, they could potentially live to become 16 years old and contribute to the production of offspring for several years, compared to individuals dying at an earlier age. This hurdle of surviving over the age of two could be tied with risks that are particularly high for younger hedgehogs. Recently published research based on the same sample set showed that the endoparasite occurrence in hedgehogs aged one year was significantly higher (n = 52/58, 90%) compared to that of hedgehogs of two years of age (n = 20/31, 65%) [108]. It may also be the case that individuals gradually gain more experience as they grow older. If the individuals have managed to survive to reach the age of two years or more, they would have likely learned to avoid dangers such as cars and predators and have established good nests and foraging routines throughout their home range [109].

## 5. Conclusions

Analysing this comprehensive dataset of the Danish population of European hedgehogs, we found that the mean age-at-death of all individuals was 1.8 years, 1.6 years for females and 2.1 years for males, through the counting of periosteal growth lines. The age-at-death ranged from 0 to 16 years, and the hedgehog reaching 16 years is currently the oldest scientifically documented individual of the species. Our actuarial life tables showed that life expectancy at age 0 (*e_x_*) was 2.6 years for males and 2.1 years for females. The results of both methods indicate that male hedgehogs live longer than females in terms of maximum recorded life span, which stands in contrast to most other mammal species. The fact that an average hedgehog reaches an approximate age of two years in Denmark means that most individuals will have the opportunity to take part in at least two breeding seasons during their lifetime.

Future work could use our results, alongside additional data on age-specific reproductive output data, to construct matrix population models that could be used for more robust modelling of European hedgehog populations. These would indicate whether or not the populations of hedgehogs are in decline, enabling an evaluation of the conservation status of the European hedgehog. However, in spite of the great concern for the populations in more recent times, we do not yet have sufficient demographic data to adequately determine the demographic health of the population through a population matrix model. We would therefore like to address a need for representative demographic data on age/stage-specific survival and fecundity to be able to assess the current demographic trends of European hedgehogs.

In conclusion, the various findings of this study have improved our understanding of the basic life history of hedgehogs in Denmark. This information, and in particular the mortality trajectories of males and females, will eventually generate improved modelling of population dynamics to inform the important conservation management for this declining species.

## Figures and Tables

**Table 1:** Overview of age determination studies on hedgehogs, including the present study. * signifies that individuals of the age group 0–1 year have been omitted from the calculation of mean age. A blank field indicates missing data. Measures of age are presented in years. Brockie [47]: Detailed information was limited. One hundred forty-three individuals, 11% (n = 16) over 18 months (counted as 1-year-olds) and 7 over 2.5 years counted as 2-year-olds. The mean value is based on 120 individuals of 0–1 years, 16 individuals of 1–2 years, and 7 individuals of 2–3 years. Brockie [47] estimated that average life expectancy from birth was around 18 months. Brockie [64] estimated that the life expectancy was less than 2.7 years and probably closer to 2 years. Calculating the life expectancy excluding the individuals below 1 year of age, the mean is 2.7 years. When including individuals younger than 1 year of age, the mean is 1.8 years.

Author	Country	Technique	Period	Sample Size			Mean Age			Age Span	
				Female	Male	**Total**	Female	Male	**Total**		
Brockie [47]	New Zealand	Tooth abrasions				**143**			**0.4 (see table text)**	0–2	
Brockie [64]	New Zealand	Mandible (periosteal growth lines)				**83**			<2.7* or 1.8 (see table text)	0–7	
Dickman [48]	United Kingdom	Mandible (periosteal growth lines)	1982–84			**87**			3.3*	1–8	
Döpke [49]	Germany	Mandible (periosteal growth lines)	1980–2001	32	34	**66**	0.8	1.7	**1.2**	0–6	
Haigh, Kelly, Butler and O’Riordan [50]	Ireland	Mandible (periosteal growth lines)	2008–2011	31	46	**83 (6 of unknown sex)**	2.1	1.9	**1.9**	0–9	
Heddergott [51]	Thüringen, Germany	Tooth growth lines	1993–2002	81	99	**185 (5 of unknown sex)**	2.5	2.1	2.3*	1–7	
Heddergott [52]	Harz, Germany	Tooth growth lines	1997–2002	49	57	**106**	1.6	1.5	1.5*	1–4	
Heddergott [53]	Germany, Saalfeld-Rudolstadt (Thüringen)	Tooth growth lines	1992–2003	44	37	**81**	1.9	2.5	2.2*	1–8	
Heddergott and Müller [54]	Germany, Osthessen	Tooth growth lines	1980–2006	62	61	**124**	1.9	1.7	1.8*	1–6	
Heddergott, Steinbach and Heddergott [55]	Germany, Heilbad Heiligenstadt (Thüringen)	Tooth growth lines	1998–2004	62	51	**113**	2.2	2.5	2.3*	1–7	
Heyne [56]	Germany	Tooth abrasions+ Tooth growth lines	1981–89			**62**			2*	1–5	
Kristoffersson [58]	Finland	Mandible (periosteal growth lines)	1960–61			**67**			2.7* (individuals < 500 g excluded)	1–7	
Scott-Hayward [68]	Uists, Outer Hebrides, UK	Mandible (periosteal growth lines)				**66**			2.6*	1–6	
Morris [36], Morris [41], Morris [60]	Southern England, United Kingdom	Mandible (periosteal growth lines)	1965–68	102	127	**244 (15 of unknown sex)**			**1.7**	0–6	
Neuschulz and Schubert [60]	Dresden-Bühlau, Germany	Capture-mark-recapture	1984–89			**125 (60 adults and 65 juveniles marked, 53 recaptured over 5 years)**			2.1*	1–6	
Parkes [65]	Manawatu, New Zealand	Capture-mark-recapture + calculation using a formula [66]	1970–1971			**144 (73 juveniles)**			**2.0**		
Rasmussen et al. 2023 (present study)	Denmark	Mandible (periosteal growth lines)	2016	109	177	**388 (101 of unknown sex)**	1.6	2.2 (1.6 for unknown sex)	**1.8**	0–16	
Rautio, Kunnasranta, Valtonen, Ikonen, Hyvarinen, Holopainen and Kukkonen [61]	Finland	Mandible (periosteal growth lines)	2004–2005	31	34	**65**	1.5	1.7	**1.6**	0–7	
Reeve, Love and Shore [42]	United Kingdom	Mandible (periosteal growth lines)	1988–89			**68**			**1.6**	0–7	
Skoudlin [62]	Czech republic	Tooth abrasions		43	63	**106**			2.5*	1–5	
Skoudlin [63]	Czech republic, Bohemina and Moravia	Tooth abrasions				**215**			**2.1**	0–6	

**Table 2:** Overview of age estimation data showing the number of individuals at each age, in years, out of a total sample of 388 hedgehogs. Age was estimated by counting the periosteal growth lines in transverse sections of the lower jaws. Rows are omitted where no individuals of that age were found.

Age in Years	Number of Individuals	Sex	Distribution by Sex
**0**	**109**		
		*Male*	43
		*Female*	39
		*Unknown*	27
**1**	**115**		
		*Male*	54
		*Female*	21
		*Unknown*	40
**2**	**54**		
		*Male*	23
		*Female*	15
		*Unknown*	16
**3**	**53**		
		*Male*	29
		*Female*	18
		*Unknown*	6
**4**	**22**		
		*Male*	6
		*Female*	9
		*Unknown*	7
**5**	**15**		
		*Male*	7
		*Female*	6
		*Unknown*	2
**6**	**11**		
		*Male*	8
		*Female*	1
		*Unknown*	2
**9**	**2**		
		*Male*	2
		*Female*	0
		*Unknown*	0
**10**	**4**		
		*Male*	2
		*Female*	0
		*Unknown*	2
**11**	**1**		
		*Male*	1
		*Female*	0
		*Unknown*	0
**13**	**1**		
		*Male*	1
		*Female*	0
		*Unknown*	0
**16**	**1**		
		*Male*	1
		*Female*	0
		*Unknown*	0
**Total individuals**	**388**		

**Table 3:** An empirical life table for male hedgehogs. Nomenclature follows Jones [73]: *x*: the exact age at the start of the interval; *n*: the length of the interval in person-years (the difference between the values of *x* in consecutive rows); *l_x_*: the number of individuals entering the interval at age *x*, with the first entry being the number of individuals in the entire cohort and subsequent entries being the number surviving to each age (*x*); *_n_d_x_*: the number of individuals dying between ages *x* and *x* + *n*; *_n_q_x_*: the probability of dying, calculated as *_n_d_x_*/*l_x_*; *_n_p_x_*: the probability of surviving, calculated as 1 − (*_n_d_x_*/*lx*);*_n_L_x_*: person-years lived between ages *x* and x + *n*; *T_x_*: person-years lived above age *x*; *e_x_*: life expectancy from age *x*; *_n_m_x_*: death rate in the cohort between ages *x* and *x* + *n*; *_n_a_x_*: the average number of years lived in the time interval by those dying in the time interval, was assumed to be 0.5 throughout.

*x*	*l_x_*	*_n_d_x_*	*_n_q_x_*	*_n_p_x_*	*_n_L_x_*	*T_x_*	*e_x_*	*_n_m_x_*	*_n_a_x_*
0	177	43	0.243	0.757	155.5	460.5	2.602	0.277	0.5
1	134	54	0.403	0.597	107.0	305.0	2.276	0.505	0.5
2	80	23	0.287	0.713	68.5	198.0	2.475	0.336	0.5
3	57	29	0.509	0.491	42.5	129.5	2.272	0.682	0.5
4	28	6	0.214	0.786	25.0	87.0	3.107	0.240	0.5
5	22	7	0.318	0.682	18.5	62.0	2.818	0.378	0.5
6	15	8	0.533	0.467	11.0	43.5	2.900	0.727	0.5
7	7	0	0.000	1.000	7.0	32.5	4.643	0.000	0.5
8	7	0	0.000	1.000	7.0	25.5	3.643	0.000	0.5
9	7	2	0.286	0.714	6.0	18.5	2.643	0.333	0.5
10	5	2	0.400	0.600	4.0	12.5	2.500	0.500	0.5
11	3	1	0.333	0.667	2.5	8.5	2.833	0.400	0.5
12	2	0	0.000	1.000	2.0	6.0	3.000	0.000	0.5
13	2	1	0.500	0.500	1.5	4.0	2.000	0.667	0.5
14	1	0	0.000	1.000	1.0	2.5	2.500	0.000	0.5
15	1	0	0.000	1.000	1.0	1.5	1.500	0.000	0.5
16	1	1	1.000	0.000	0.5	0.5	0.500	2.000	0.5
17	0	0	-	-	-	0.0	-	-	0.5

**Table 4:** An empirical life table for female hedgehogs. Nomenclature follows Jones [73]: x: the exact age at the start of the interval; *n*: the length of the interval in person-years (the difference between the values of *x* in consecutive rows); *l_x_*: the number of individuals entering the interval at age *x*, with the first entry being the number of individuals in the entire cohort and subsequent entries being the number surviving to each age (*x*); *_n_d_x_*: the number of individuals dying between ages *x* and *x* + *n*; *_n_q_x_*: the probability of dying, calculated as *_n_d_x_*/*l_x_*; *_n_p_x_*: the probability of surviving, calculated as 1 − (*_n_d_x_*/*lx*);*_n_L_x_*: person-years lived between ages *x* and x + *n*; *T_x_*: person-years lived above age *x*; *e_x_*: life expectancy from age *x*; *_n_m_x_*: death rate in the cohort between ages *x* and *x* + *n*; *_n_a_x_*: the average number of years lived in the time interval by those dying in the time interval, was assumed to be 0.5 throughout.

*x*	*l_x_*	*_n_d_x_*	*_n_q_x_*	*_n_p_x_*	*_n_L_x_*	*T_x_*	*e_x_*	*_n_m_x_*	*_n_a_x_*
0	109	39	0.358	0.642	89.5	231.5	2.124	0.436	0.5
1	70	21	0.300	0.700	59.5	142.0	2.029	0.353	0.5
2	49	15	0.306	0.694	41.5	82.5	1.684	0.361	0.5
3	34	18	0.529	0.471	25.0	41.0	1.206	0.720	0.5
4	16	9	0.562	0.438	11.5	16.0	1.000	0.783	0.5
5	7	6	0.857	0.143	4.0	4.5	0.643	1.500	0.5
6	1	1	1.000	0.000	0.5	0.5	0.500	2.000	0.5
7	0	0	-	-	-	0.0	-	-	0.5

## Data Availability

The dataset is available from Zenodo (https://doi.org/10.5281/zenodo.7615782).

## References

[1] Roff D.A. (2002). Life History Evolution.

[2] Roff D. (1992). Evolution of Life Histories: Theory and Analysis.

[3] Reznick D.A., Bryga H., Endler J.A. (1990). Experimentally induced life-history evolution in a natural population. Nature.

[4] Neal D. (2003). Introduction to Population Biology.

[5] Spencer W., Rustigian-Romsos H., Strittholt J., Scheller R., Zielinski W., Truex R. (2011). Using occupancy and population models to assess habitat conservation opportunities for an isolated carnivore population. Biol. Conserv..

[6] Tempel D.J., Peery M., Gutierrez R.J. (2014). Using integrated population models to improve conservation monitoring: California spotted owls as a case study. Ecol. Model..

[7] Sierra Forest Legacy Pacific fisher (Pekania pennanti). https://www.sierraforestlegacy.org/FC_SierraNevadaWildlifeRisk/PacificFisher.php.

[8] Sierra Forest Legacy California Spotted Owl (Strix occidentalis occidentalis). https://www.sierraforestlegacy.org/FC_SierraNevadaWildlifeRisk/CaliforniaSpottedOwl.php.

[9] Morris P. (2014). Hedgehogs.

[10] Reeve N. (1994). Hedgehogs.

[11] Müller F. (2018). Langzeit-Monitoring der Strassenverkehrsopfer beim Igel (*Erinaceus europaeus L.*) zur Indikation von Populationsdichteveränderungen entlang zweier Teststrecken im Landkreis Fulda. Beiträge Nat. Osthess..

[12] SoBH (2011). The state of Britain’s Hedgehogs 2011.

[13] SoBH (2015). The State of Britain’s Hedgehogs 2015.

[14] SoBH (2018). The State of Britain’s Hedgehogs 2018.

[15] Hof A.R., Bright P.W. (2016). Quantifying the long-term decline of the West European hedgehog in England by subsampling citizen-science datasets. Eur. J. Wildl. Res..

[16] Krange M. (2015). Change in the occurrence of the West European Hedgehog (Erinaceus europaeus) in western Sweden during 1950–2010. Ph.D. Thesis.

[17] van de Poel J.L., Dekker J., van Langevelde F. (2015). Dutch hedgehogs *Erinaceus europaeus* are nowadays mainly found in urban areas, possibly due to the negative effects of badgers Meles meles. Wildl. Biol..

[18] Williams B.M., Baker P.J., Thomas E., Wilson G., Judge J., Yarnell R.W. (2018). Reduced occupancy of hedgehogs (*Erinaceus europaeus*) in rural England and Wales: The influence of habitat and an asymmetric intra-guild predator. Sci. Rep..

[19] Taucher A.L., Gloor S., Dietrich A., Geiger M., Hegglin D., Bontadina F. (2020). Decline in Distribution and Abundance: Urban Hedgehogs under Pressure. Animals.

[20] Wembridge D., Johnson G., Al-Fulaij N., Langton S. (2022). The State of Britain’s Hedgehogs 2022.

[21] Mathews F., Harrower C. IUCN—Compliant Red List for Britain’s Terrestrial Mammals. Assessment by the Mammal Society under contract to Natural England, Natural Resources Wales and Scottish Natural Heritage.

[22] Brakes C.R., Smith R.H. (2005). Exposure of non-target small mammals to rodenticides: Short-term effects, recovery and implications for secondary poisoning. J. Appl. Ecol..

[23] Haigh A., O’Riordan R.M., Butler F. (2012). Nesting behaviour and seasonal body mass changes in a rural Irish population of the Western hedgehog (*Erinaceus europaeus*). Acta Theriol..

[24] Hof A.R., Bright P.W. (2010). The value of agri-environment schemes for macro-invertebrate feeders: Hedgehogs on arable farms in Britain. Anim. Conserv..

[25] Huijser M.P., Bergers P.J.M. (2000). The effect of roads and traffic on hedgehog (*Erinaceus europaeus*) populations. Biol. Conserv..

[26] Young R.P., Davison J., Trewby I.D., Wilson G.J., Delahay R.J., Doncaster C.P. (2006). Abundance of hedgehogs (*Erinaceus europaeus*) in relation to the density and distribution of badgers (*Meles meles*). J. Zool..

[27] Hubert P., Julliard R., Biagianti S., Poulle M.-L. (2011). Ecological factors driving the higher hedgehog (*Erinaceus europeaus*) density in an urban area compared to the adjacent rural area. Landsc. Urban Plan..

[28] Pettett C.E., Moorhouse T.P., Johnson P.J., Macdonald D.W. (2017). Factors affecting hedgehog (Erinaceus europaeus) attraction to rural villages in arable landscapes. Eur. J. Wildl. Res..

[29] Dowding C.V., Harris S., Poulton S., Baker P.J. (2010). Nocturnal ranging behaviour of urban hedgehogs, *Erinaceus europaeus*, in relation to risk and reward. Anim. Behav..

[30] Dowding C.V., Shore R.F., Worgan A., Baker P.J., Harris S. (2010). Accumulation of anticoagulant rodenticides in a non-target insectivore, the European hedgehog (*Erinaceus europaeus*). Environ. Pollut..

[31] Rasmussen S.L., Nielsen J.L., Jones O.R., Berg T.B., Pertoldi C. (2020). Genetic structure of the European hedgehog (*Erinaceus europaeus*) in Denmark. PLoS ONE.

[32] Rasmussen S.L., Yashiro E., Sverrisdóttir E., Nielsen K.L., Lukassen M.B., Nielsen J.L., Asp T., Pertoldi C. (2019). Applying the GBS Technique for the Genomic Characterization of a Danish Population of European Hedgehogs (*Erinaceus europaeus*). Genet. Biodivers. (GABJ).

[33] Rasmussen S.L., Berg T.B., Dabelsteen T., Jones O.R. (2019). The ecology of suburban juvenile European hedgehogs (*Erinaceus europaeus*) in Denmark. Ecol. Evol..

[34] Burroughes N.D., Dowler J., Burroughes G. (2021). Admission and Survival Trends in Hedgehogs Admitted to RSPCA Wildlife Rehabilitation Centres. Proc. Zool. Soc..

[35] Lukešová G., Voslarova E., Vecerek V., Vucinic M. (2021). Trends in intake and outcomes for European hedgehog (*Erinaceus europaeus*) in the Czech rescue centers. PLoS ONE.

[36] Morris P. (2018). Hedgehog.

[37] Rasmussen S.L., Kalliokoski O., Dabelsteen T., Abelson K. (2021). An exploratory investigation of glucocorticoids, personality and survival rates in wild and rehabilitated hedgehogs (*Erinaceus europaeus*) in Denmark. BMC Ecol. Evol..

[38] Jensen A.B. (2004). Overwintering of European hedgehogs *Erinaceus europaeus* in a Danish rural area. Acta Theriol..

[39] Park E.A. (1964). The imprinting of nutritional disturbances on the growing bone. Pediatrics.

[40] Peabody F.E. (1961). Annual growth zones in living and fossil vertebrates. J. Morphol..

[41] Morris P.A. (1970). A method for determining absolute age in the hedgehog. J. Zool..

[42] Reeve N.J., Love B., Shore R. (1996). X-ray measures of long bones as an aid to age-determination in hedgehogs (*Erinaceus europaeus*). Proceedings of the 1st European Hedgehog Research Group Meeting.

[43] Ohtaishi N., Hachiya N., Shibata Y. (1976). Age determination of the hare from annual layers in the mandibular bone. Acta Theriol..

[44] Henderson B.A., Bowen H.M. (1979). Estimating the age of the European rabbit, *Oryctolagus Cuniculus*, by counting the adhesion lines in the periosteal zone of the lower mandible. J. Appl. Ecol..

[45] Castanet J., Croci S., Aujard F., Perret M., Cubo J., de Margerie E. (2004). Lines of arrested growth in bone and age estimation in a small primate: *Microcebus murinus*. J. Zool..

[46] Sander P.M., Andrássy P. (2006). Lines of arrested growth and long bone histology in Pleistocene large mammals from Germany: What do they tell us about dinosaur physiology?. Palaeontogr. Abt. A.

[47] Brockie R.E. (1958). Ecology of the hedgehog *Erinaceus europaeus* in New Zealand. Master’s Thesis.

[48] Dickman C.R. (1988). Age-related dietary change in the European hedgehog, *Erinaceus europaeus*. J. Zool..

[49] Döpke C. (2002). Kasuistische Auswertung der Untersuchungen von Igeln (*Erinaceus europaeus*) im Einsendungsmaterial des Instituts für Pathologie von 1980 bis 2001. Ph.D. Thesis.

[50] Haigh A., Kelly M., Butler F., O’Riordan R.M. (2014). Non-invasive methods of separating hedgehog (*Erinaceus europaeus*) age classes and an investigation into the age structure of road kill. Acta Theriol..

[51] Heddergott M. (2003). Zur Altersstruktur des Igels *Erinaceus europaeus* (L., 1758) in Thüringen anhand von Schädeln (Mammalia: Insectivora, Erinaceidae). Veröffentlichungen Naturhist. Mus. Schleus..

[52] Heddergott M. (2004). Age-structure of hedgehog *Erinaceus europaeus* (L., 1758) in the Harz Mountains. Abh. Und Ber. Aus Dem Mus. Heine..

[53] Heddergott M. (2005). Age structure of the hedgehog *Erinaceus europaeus* L., 1758, in the district Saalfeld-Rudolstadt (Thuringia). Hercynia.

[54] Heddergott M., Müller F. (2008). Zur Altersstruktur zweier Populationen des Braunbrustigels *Erinaceus europaeus L.*, 1758 (Mammalia: Insectivora) in Osthessen. Beiträge Zur Nat. Osthess..

[55] Heddergott M., Steinbach O., Heddergott C. (2010). Zur Altersstruktur des Braunbrustigels *Erinaceus europaeus* (Linnaeus, 1758) im Stadtgebiet Heilbad Heiligenstadt (Thüringen) (Mammalia: Insectivora, Erinaceidae)*. Mauritiana.

[56] Heyne P. (1990). Beitrag zur Altersstruktur von Erinaceus europaeus L., 1758. Populationsökologie von Kleinsäugerarten, Martin-Luther-Universitaet Halle-Wittenberg Wissenschaftliche Beitrage. SLUB.

[57] Kratochvil J. (1975). Hedgehogs of the genus *Erinaceus* in Czechoslovakia insectivora mammalia. Zool. Listy.

[58] Kristoffersson R. (1971). A note on the age distribution of hedgehogs *Erinaceus europaeus* in Finland. Ann. Zool. Fenn..

[59] Morris P.A. (1969). Some Aspects of the Ecology of the Hedgehog (*Erinaceus europaeus*).

[60] Neuschulz N., Schubert M. (1990). Altersermittlung bei *Erinaceus europaeus L.,* 1758 an einer Igelfütterung. Popul. Von Kleinsäugerarten Wiss. Beitr. Univ. Halle.

[61] Rautio A., Kunnasranta M., Valtonen A., Ikonen M., Hyvarinen H., Holopainen I.J., Kukkonen J.V.K. (2010). Sex, Age, and Tissue Specific Accumulation of Eight Metals, Arsenic, and Selenium in the European Hedgehog (*Erinaceus europaeus*). Arch. Environ. Contam. Toxicol..

[62] Skoudlin J. (1976). On determining the age in erinaceus-europaeus and erinaceus-concolor insectivora erinaceidae. Vestn. Ceskoslovenske Spol. Zool..

[63] Skoudlin J. (1981). Age structure of czechoslovak populations of Erinaceus-Europaeus and Erinaceus-Concolor Insectivora Erinaceidae. Vestn. Ceskoslovenske Spol. Zool..

[64] Brockie R.E. (1974). Studies of the Hedgehog Erinaceus europaeus L. in New Zealand.

[65] Parkes J. (1975). Some aspects of the biology of the hedgehog erinaceus-europaeus in the manawatu new-zealand. New Zealand J. Zool..

[66] Petrides G.A. (1951). The determination of sex and age ratios in the cottontail rabbit. Am. Midl. Nat..

[67] Morris P.A. (1971). Epiphyseal fusion in the forefoot as a means of age determination in the hedgehog (*Erinaceus europaeus*) *J*. Zool..

[68] Scott-Hayward L., Morris P.A. (2018). Data on age determination (pers. comm. p. 224). Hedgehogs.

[69] R Core Team (2022). A Language and Environment for Statistical Computing.

[70] Hijmans R.J., raster: Geographic Data Analysis and Modeling R package. https://cran.r-project.org/web/packages/raster/index.html.

[71] Rasmussen S.L., Larsen J., van Wijk R.E., Jones O.R., Berg T.B., Angen O., Larsen A.R. (2019). European hedgehogs (*Erinaceus europaeus*) as a natural reservoir of methicillin-resistant Staphylococcus aureus carrying mecC in Denmark. PLoS ONE.

[72] Samuel Preston P.H., Guillot M. (2020). Demography: Measuring and Modeling Population Processes.

[73] Jones O.R. (2021). Life tables: Construction and interpretation In Demographic Methods Across the Tree of Life.

[74] Kulus M.J., Dąbrowski P. (2019). How to calculate the age at formation of Harris lines? A step-by-step review of current methods and a proposal for modifications to Byers’ formulas. Archaeol. Anthropol. Sci..

[75] Foster N.J., Maloney R.F., Recio M.R., Seddon P.J., van Heezik Y. (2021). European hedgehogs rear young and enter hibernation in New Zealand’s alpine zones. New Zealand J. Ecol..

[76] Walhovd H. (1976). Winter activity of Danish hedgehogs in 1973-74 with information on the size of the animals observed and location of the recordings. Flora Og Fauna.

[77] Walhovd H. (1978). The overwintering pattern of Danish hedgehogs in outdoor confinement, during three successive winters. Nat. Jutl..

[78] Hansen L. (1982). Trafikdræbte dyr i Danmark. Dan. Ornitol. Foren. Tidsskr..

[79] Holsbeek L., Rodts J., Muyldermans S. (1999). Hedgehog and other animal traffic victims in Belgium: Results of a countrywide survey. Lutra.

[80] Schwartz A.L., Shilling F.M., Perkins S.E. (2020). The value of monitoring wildlife roadkill. Eur. J. Wildl. Res..

[81] Wembridge D., Newman M.R., Bright P., Morris P. (2016). An estimate of the annual number of hedgehog (Erinaceus europaeus) road casualties in Great Britain.

[82] Kent E., Schwartz A.L., Perkins S.E. (2021). Life in the fast lane: Roadkill risk along an urban–rural gradient. J. Urban Ecol..

[83] Raynaud J., Schradin C. (2014). Experimental increase of testosterone increases boldness and decreases anxiety in male African striped mouse helpers. Physiol. Behav..

[84] Morris P.A. (1988). A study of home range and movements in the hedgehog (*Erinaceus europaeus*). J. Zool..

[85] Reeve N.J. (1982). The home range of the hedgehog as revealed by a radio tracking study. Symp. Zool. Soc. Lond..

[86] Kristiansson H. (1984). Ecology of a hedgehog (*Erinaceus europaeus*) population in Southern Sweden. PhD Thesis.

[87] Haigh A., O’Riordan R.M., Butler F. (2014). Hedgehog *Erinaceus europaeus* mortality on Irish roads. Wildl. Biol..

[88] Göransson G., Karlsson J., Lindgren A. (1976). Road mortality of the hedgehog *Erinaceus europaeus* in southern Sweden (Igelkotten och biltrafiken). Fauna Och Flora.

[89] Huijser M.P. (2000). Life on the Edge: Hedgehog Traffic Victims and Mitigation Strategies in an Anthropogenic Landscape.

[90] Vejdirektoratet Trafik på strækninger 2021. http://vej08.vd.dk/stroemkort/nytui/kort/Stroemkort.html?id=201.

[91] Rondinini C., Doncaster C.P. (2002). Roads as barriers to movement for hedgehogs. Funct. Ecol..

[92] Brockie R.E., Sadleir R.M., Linklater W.L. (2009). Long-term wildlife road-kill counts in New Zealand. New Zealand J. Zool..

[93] Moore L.J., Petrovan S.O., Baker P.J., Bates A.J., Hicks H.L., Perkins S.E., Yarnell R.W. (2020). Impacts and potential mitigation of road mortality for hedgehogs in Europe. Animals.

[94] Slater F.M. (2002). An assessment of wildlife road casulties—the potential discrepancy between numbers counted and numbers killed. Web Ecol..

[95] Ratton P., Secco H., Da Rosa C.A. (2014). Carcass permanency time and its implications to the roadkill data. Eur. J. Wildl. Res..

[96] Schwartz A.L.W., Williams H.F., Chadwick E., Thomas R.J., Perkins S.E. (2018). Roadkill scavenging behaviour in an urban environment. J. Urban Ecol..

[97] Keller L.F., Waller D.M. (2002). Inbreeding effects in wild populations. Trends Ecol. Evol..

[98] Wright P.G. (2020). , Coomber, F. G., Bellamy, C. C., Perkins, S. E., Mathews, F. Predicting hedgehog mortality risks on British roads using habitat suitability modelling. PeerJ.

[99] Austad S.N. (2006). Why women live longer than men: Sex differences in longevity. Gend. Med..

[100] Clutton-Brock T.H., Isvaran K. (2007). Sex Differences in Ageing in Natural Populations of Vertebrates. Proc. Biol. Sci..

[101] Trivers R.L., Campbell B. (1972). Parental investment and sexual selection. Sexual Selection and the Descent of Man.

[102] Beirne C., Delahay R., Young A. (2015). Sex differences in senescence: The role of intra-sexual competition in early adulthood. Proc. R. Soc. B Biol. Sci..

[103] Dugdale H.L., Macdonald D.W., Pope L.C., Burke T. (2007). Polygynandry, extra-group paternity and multiple-paternity litters in European badger (*Meles meles*) social groups. Mol. Ecol..

[104] Delahay R., Walker N., Forrester G., Harmsen B., Riordan P., Macdonald D., Newman C., Cheeseman C. (2006). Demographic correlates of bite wounding in Eurasian badgers, *Meles meles* L., in stable and perturbed populations. Anim. Behav..

[105] Macdonald D.W., Newman C., Nouvellet P.M., Buesching C.D. (2009). An Analysis of Eurasian Badger (*Meles meles*) Population Dynamics: Implications for Regulatory Mechanisms. J. Mammal..

[106] Sugianto N.A., Newman C., Macdonald D.W., Buesching C.D. (2020). Reproductive and somatic senescence in the European badger (*Meles meles*): Evidence from lifetime sex-steroid profiles. Zoology.

[107] Woodroffe R. (1993). Alloparental behaviour in the European badger. Anim. Behav..

[108] Rasmussen S.L., Hallig J., van Wijk R.E., Petersen H.H. (2021). An investigation of endoparasites and the determinants of parasite infection in European hedgehogs (*Erinaceus europaeus*) from Denmark. Int. J. Parasitol. Parasites Wildl..

[109] Marshall H.H., Vitikainen E.I., Mwanguhya F., Businge R., Kyabulima S., Hares M.C., Inzani E., Kalema-Zikusoka G., Mwesige K., Nichols H.J. (2017). Lifetime fitness consequences of early-life ecological hardship in a wild mammal population. Ecol. Evol..

